# Pooled samples and eDNA-based detection can facilitate the “clean trade” of aquatic animals

**DOI:** 10.1038/s41598-020-66280-7

**Published:** 2020-06-24

**Authors:** Jesse L. Brunner

**Affiliations:** Washington State University, School of Biological Sciences, Pullman, WA 99164 USA

**Keywords:** Statistical methods, Ecological epidemiology, Epidemiology

## Abstract

The regional and international trade of live animals facilitates the movement, spillover, and emergence of zoonotic and epizootic pathogens around the world. Detecting pathogens in trade is critical for preventing their continued movement and introduction, but screening a sufficient fraction to ensure rare infections are detected is simply infeasible for many taxa and settings because of the vast numbers of animals involved—hundreds of millions of live animals are imported into the U.S.A. alone every year. Batch processing pools of individual samples or using environmental DNA (eDNA)—the genetic material shed into an organism’s environment—collected from whole consignments of animals may substantially reduce the time and cost associated with pathogen surveillance. Both approaches, however, lack a framework with which to determine sampling requirements and interpret results. Here I present formulae for pooled individual samples (e.g,. swabs) and eDNA samples collected from finite populations and discuss key assumptions and considerations for their use with a focus on detecting *Batrachochytrium salamandrivorans*, an emerging pathogen that threatens global salamander diversity. While empirical validation is key, these formulae illustrate the potential for eDNA-based detection in particular to reduce sample sizes and help bring clean trade into reach for a greater number of taxa, places, and contexts.

## Introduction

The regional and international trade in live animals facilitates the inadvertent translocation, spillover, and emergence of zoonotic and epizootic pathogens around the world^1–7^. From the spread of agricultural pathogens (e.g., African swine fever virus, foot and mouth disease virus, or rinderpest virus) to emerging zoonoses (e.g., Avian influenza virus, SARS-CoV-2), “pathogen pollution”^1,2^ can have dramatic socioeconomic impacts and threaten human health^5,8–11^. It is also playing an increasingly important role in wildlife conservation^1,2,9,12,13^. Once established, pathogens are usually difficult if not impossible to eradicate or even control^14–16^. Thus, detecting pathogens in trade in order to prevent their introduction and establishment is a key goal^6,9,17–19^.

A primary challenge for routine surveillance of the live animal trade is its magnitude. The pathways of trade are diverse and complex^6,10^ so only portions are well characterized, but two estimates are illustrative and sobering. First, over a billion ornamental fish are traded internationally every year^14^. Second, the USA alone imports an average of 225 million individuals in addition to 1.8 × 10^6^ kg of live animals per year, the “vast majority” of which are part of the aquatics and pet industry^6^. While there are national or international regulations governing the trade of certain taxa (e.g., those of economic importance, threats to human health, threatened and endangered species), for most there is little on-the-ground scrutiny and little or no surveillance for disease. (Australia, with its strong focus on biosecurity, is an admirable exception^20^.) Indeed, detecting infections with standard, individual-based sampling—that is, collecting a swab, tissue, or blood sample from each individual for subsequent diagnostic tests—requires such large sample sizes as to frequently make routine surveillance uneconomical, politically unpalatable, or simply infeasible. Even under a best-case scenario (e.g., using a diagnostically perfect test to detect a pathogen occurring at 10% prevalence) one would still need to screen 25 individuals in a consignment of 100 animals; multiplied by the myriad consignments (e.g., >10^5^ per year into the USA alone^6^) and the number becomes daunting. More often we are interested in detecting rare infections with imperfect diagnostic tests (i.e., false negatives and false positives occur at some non-trivial rate) and the sample sizes increase concomitantly.

Two approaches have been suggested to reduce the numbers of samples to more manageable levels. First, individual samples can be pooled and processed as a group^21–23^, reducing the level of sample processing (e.g., DNA extractions) and screening (e.g., PCR reactions, ELISAs) several-fold. First proposed by Dorfman^21^ in 1943 in the context of screening U.S. service members for syphilis, pooling samples to reduce the number of tests required to detect rare infections is common in numerous contexts^22^, including surveys for disease freedom. However, the formulae for inference from pooled samples generally assume sampling with replacement or, equivalently, very large populations, which does not align well with the problem of detecting infections in consignments or captive populations. Recently a probability formula was developed for pooled samples collected from finite populations without replacement^24^—but it does not accommodate imperfect tests.

Second, one might screen environmental DNA (eDNA)—the genetic material shed into an organism’s environment—for the pathogen or parasite of interest^25^. The eDNA approach seems especially relevant to aquatic and semi-aquatic taxa, which make up a large portion of the wildlife trade^6^, as eDNA can be simply filtered from water housing animals; they need not even be handled. (Semi-aquatic species or life stages are often shipped in moist sphagnum moss or paper, but can then be placed in water to collect eDNA during a quarantine period^26^.) Environmental DNA sampling is being used to detect pathogens in a growing number of settings, from natural environments^27–30^ to ballast water^31^ and even in trade^32–34^. However, while the statistical framework for making proper inference from eDNA results is well-developed in certain contexts (e.g., the presence or absence of a target species in ponds using occupancy models^29,35^), these approaches do not translate well to sampling small captive populations or consignments.

While both approaches have the potential to reduce sample sizes, we lack the formal framework with which to determine sampling requirements and interpret results. In this paper I therefore develop formulae for imperfect tests of pooled samples in closed populations and eDNA, discuss the key assumptions and considerations in their application, and illustrate how eDNA may be especially useful for detecting infections in the live animal trade. I present these results in the context of emerging fungal pathogens that threaten amphibian diversity, for which trade appears to play a key role.

## Chytrid fungi as an exemplar

The amphibian chytrid fungus, *Batrachochytrium dendrobatidis* (*Bd*), is the most devastating emerging pathogen on record, responsible for declines in over 500 species, including 90 that are likely extinct^36^. It likely owes its global distribution in large measure to the international trade of African clawed frogs (*Xenopus laevis*) used for pregnancy tests and research^37^, American bullfrogs (*Lithobates catesbeianus*) sold for food^38,39^, and myriad species involved in the pet trade^33,40–43^. More recently, the international pet trade aided the emergence of a novel chytrid fungus, *B. salamandrivorans* (*Bsal*), that is particularly lethal to salamanders^44–47^.

*Bsal* was introduced into Northern Europe via the pet trade from Southeast Asia^46,48–50^. While it has not yet been detected in the wild in much of Europe^51–54^, it is already prevalent in and appears to have spread among private collections in Europe^55–57^. In North America, a hot-spot of salamander diversity, *Bsal* is apparently absent from both wild and captive amphibians^53,58–60^. However, the risk and potentially devastating consequences of its introduction via trade^61–63^ led to prohibitions on the importation of 201 species of salamanders into the U.S.A.^64^ and all salamanders in Canada^65^.

While laudable, such bans are inevitably crude. These restrictions were necessarily based on experiments with small samples sizes of few species, extended to others by taxonomic affiliation^46^. Moreover, it was quickly out of date since several other taxa, including frogs, are now known to carry *Bsal*^47,49^. Such bans may also promote black-market trade, which already occurs on a global scale similar to the black-market trade of narcotics^10^. In response to these challenges, as well as the economic interests of the pet trade, there have been calls for a “clean trade” of amphibians^66–68^. Indeed, the European Union recently mandated a program requiring screening, using a quantitative real-time PCR (qPCR) assay of skin swabs^69^, or prophylactically treating all consignments of live salamanders into and between member nations of the EU^70^.

**Figure 1:**
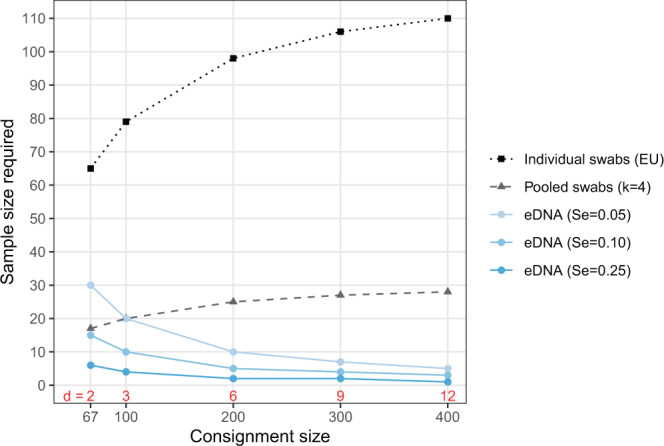
Sample sizes required to achieve a 95% chance of detecting at least one *Bsal* infection in captive populations or consignments of various sizes using individual swabs (based on EU regulations^70^), pools of four swabs, or eDNA. Calculations are based on a diagnostic sensitivity of 0.8 for swabs and a sensitivity 0.05–0.25 for eDNA, and a *Bsal* prevalence of 3% in trade^49^. The number of infected animals, *d*, in a consignment given a prevalence of 3% are shown in red at the bottom of the figure. Note that the EU regulations require testing *all* individuals in a consignment of up to 62 individuals^70^.

Again, the scale of the international trade of live amphibians makes routine screening a formidable challenge. While the number of consignments of amphibians into or between EU member nations is not available in the literature, an estimated 3–5 million live amphibians are imported per year into the USA, alone^39,71^ and 130,000 annually into the U.K.^72^. Since *Bsal* is thought to occur at low prevalence in traded animals^49^, the EU regulations require screening all or a substantial fraction of individuals in a consignment to ensure a reasonable chance of detection (Fig. 1). While shipments of certain taxa or from certain sources might be excluded, even conservative assumptions about the volume of trade imply screening an enormous number of samples if this program were extended worldwide (e.g., 1000 consignments of 200 salamanders each would require testing some 98,000 swabs). This scale of sampling is likely to place clean trade beyond the reach of many nations or interested parties (e.g., constituents in the pet trade industry). Similar problems arise when considering other amphibian pathogens (e.g., *Ranavirus*^39^) or those of fishes. The question in this paper is therefore: can pooling swab samples or using eDNA place routine surveillance in reach?

## Results

The formulae presented below can account for false positives (i.e., less than perfect specificity, *Sp*). It will be important to consider the sources and likelihood of false positives (e.g., from carry over of target DNA from the water rather than infected hosts^34,73^ vs. from contamination in a laboratory) as well as the consequences (e.g., slowing shipments, economic costs, loss of trust^32,34^). In the case of *Bsal* detection, I would hope that any positive test would be investigated further with additional, independent diagnostic tests. Moreover, setting aside the issue of carry over contamination^34^, which might be avoided by transferring animals to clean water prior to sampling, there is no evidence that false positive rates are higher in pools of samples or eDNA than typical individual samples. In fact, to the extent that fewer tests are conducted with pooling or eDNA one would expect *fewer* false positives at a given level of surveillance sensitivity. For these reasons I set aside the issue of specificity in the following discussion and focus on sensitivity.

### Pooling individual samples

I extended the “pooled hypergeometric” distribution of Theobald and Davie^24^ to account for imperfect diagnostic tests (Eq. (6)). The results are largely intuitive: when *n* individual-level samples are divided into *m* = *n*/*k* pools of size *k*, the number of pools that need be screened is reduced up to *k*-fold, assuming low prevalence of infection and high diagnostic sensitivity^24,74^. The gains in efficiency from pooling are reduced somewhat for less sensitive tests when infections are more common, but not markedly so.

A key assumption of this formula is that diagnostic sensitivity is not affected by pooling. This implies on the one hand that the combined analyte (e.g., target DNA) from multiple infected individuals does not increase diagnostic sensitivity. The probability of detecting a single infection in a pool of samples (i.e., swabs) is at most the diagnostic sensitivity of individual swabs. On the other hand, the formula assumes that the analyte is not overwhelmed or inhibited by non-target materials (e.g., PCR inhibitors in skin secretions, microbial DNA) nor diluted below a detection threshold (e.g., as was observed with pools of fish screened for a Megalocytivirus^75^). This later assumption is unlikely to hold with very large pools of samples (see Laurin *et al*.^74^ for a review), but may be reasonable for small pools. It is an important assumption to test under realistic conditions, but one could simply use lower values of diagnostic sensitivity (*Se* in Eq. (6)) to account for target swamping or dilution. Also, it is worth remembering that the same number of samples must be collected with or without pooling; efficiencies are only gained in the processing and screening steps.

### Environmental DNA

I developed a new formula for eDNA-based detection in small, closed populations (Eq. (9)). The formula makes two key assumptions. First, it assumes that the eDNA shed into the water is homogeneously distributed in the water. While pathogen eDNA is likely clumped (e.g., *Bsal* may be found principally in skin sheds), one could homogenize the water (e.g., with a blender) prior to taking samples to meet this assumption, at least in the smaller volumes used to house animals in shipments or captive populations. Thus, replicate eDNA samples are essentially technical replicates in this framework. Second, as with the formula for pooled samples, it assumes that test sensitivity is unaffected by the number of hosts in the water at the same time. That is, the target pathogen eDNA is not swamped by non-target host and microbial eDNA or other waste material leading to inhibition of the PCR reaction. This assumption needs to be empirically verified, but it may be possible to minimize PCR inhibition with dedicated kits^29^ or by diluting the eluted DNA prior to PCR, although such methods risk reducing sensitivity. In any case, the formula I present could be extended to account for this swamping effect if needed.

**Figure 2:**
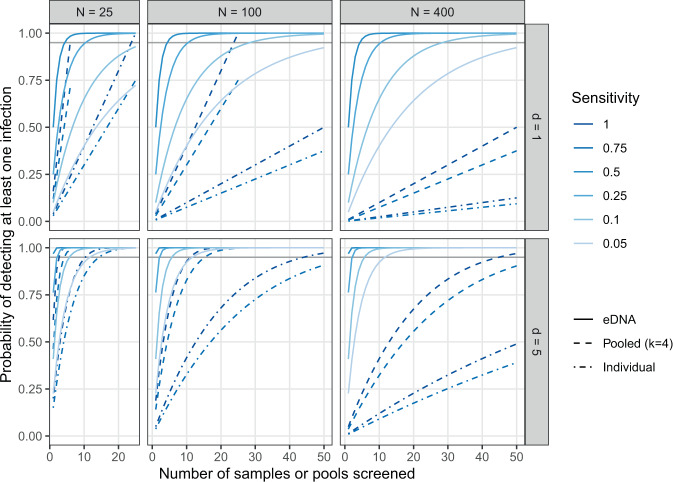
The probability of detecting at least one infection with increasing numbers of samples (or pools) screened, taken without replacement, with *d* = 1 or 5 infected individuals in a population of *N* = 25, 100, or 400. Horizontal gray lines correspond to 95% chance of detection. Note that the maximum sensitivity of eDNA-based tests shown (*Se* = 0.5) is less than the minimum sensitivity presented for individual-based tests (*Se* = 0.75). Specificity is assumed to be 1 in all cases. In contrast with Fig. 1, prevalence of infection declines with population size since the number of infected, rather than prevalence, is held constant.

These assumptions have two important consequences for eDNA-based pathogen detection. First, every sample collects eDNA from the *entire* population. Environmental DNA avoids the issue of whether rare infections are included in any random sample of individuals—they are all sampled because the eDNA from all individuals is distributed homogeneously in the water. The question is only whether there is sufficient target eDNA in a sample to ensure a reasonable probability of a positive test. As a consequence, eDNA sampling can detect rare infections with many fewer samples than individual swabs, or pools of swabs, even when diagnostic sensitivity is very low (Figs. 1 and 2). (I consider various factors that are likely to affect sensitivity in the next section.) This also means that the probability of detecting an infection is, all else being equal, independent of population size (Fig. 2). Thus, while individual-level swabbing can be more efficient than eDNA in small populations, in larger populations eDNA outperformed swabbing, even when pooled, over a wide range of parameter values (Fig. 2). Similarly, samples sizes for eDNA-based detection *decrease* with population size when prevalence is held constant because there are more infected individuals shedding pathogen eDNA, whereas they must increase with population size when using individual or pooled sampling to ensure infected individuals are included in any sample (Fig. 1). Once again, it is essential to establish the actual performance of eDNA-based detection in real world contexts and test how it scales with population size and other variables.

### Quantitative nature of detection

**Figure 3:**
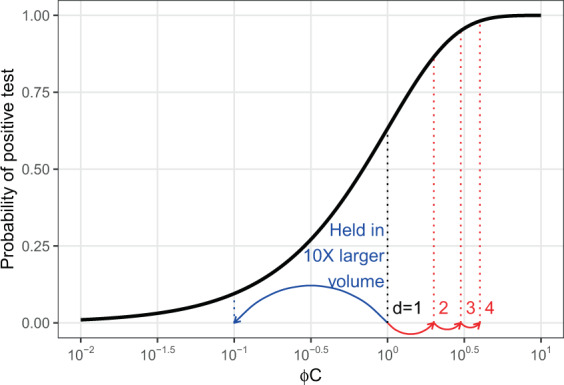
Probability of a positive test result (e.g., with a PCR reaction) as a function (Eq. (11)) of the per target copy detection rate, *ϕ* and the number of copies of the target DNA or analyte, *C*. The arrows and dotted vertical lines show the consequences of increasing the volume of water in which animals are held ten-fold, thus diluting the analyte (blue) or increasing the number of infected animals from one to two, three, or four. Note, however, that the concrete effect of such changes on the probability of a positive test depends on where one starts along the *ϕC* axis.

Diagnostic sensitivity is usually defined in the context of a classification problem with binary infection status determined by a gold standard of infection (e.g., histopathology) or assigned in experiments. As such, sensitivity is generally assumed to be fixed; that is, all infections within a population or experiment are equally detectable. However, any test that reacts directly with the pathogen (e.g., pathogen DNA or antigens) or host responses (e.g., antibodies) is necessarily dose-dependent (Fig. 3; Eq. (11)). The variation in the amount of the analyte between infected individuals or among laboratories, protocols, etc., may be small enough to ignore (but see e.g., Rimmer *et al*.^76^), or the concentrations generally large enough that detection is assured (e.g., with clearly diseased animals), in which case it may be reasonable to assume diagnostic sensitivity is constant. At the other extreme, recently infected individuals, sub-clinical carriers^16^, or otherwise inapparent infections tend to produce or shed little analyte and are thus much less easily detected than clinically infected animals. Quantitative real time PCR of swabs, for instance, often fails to detect low-level *Bd* infections^77^. Overall, variation in the status or intensity of infections among individuals (or sampling protocols) can make actual diagnostic sensitivity quite variable. Indeed, the variability among the component samples of a pool can have large effects on the pool-wide sensitivity^74^. However, the the concentration-dependent nature of detection will be especially pronounced with eDNA sampling.

The concentration of target DNA in an eDNA sample co-varies with the type and intensity of infection and details of the sampling method (here volume sampled, pore size, storage conditions^78^), as with individual samples^26^, but also depends on the conditions in which animals are held (e.g., temperature, volume, time in the water; Fig. 3). Thus, one would expect a great deal more variability in sensitivity of eDNA between settings and studies, not to mention between taxa, than with traditional sampling directly from animals. Empirical validation of eDNA-based pathogen detection should thus focus on understanding the causes and consequences of this variability across consignment types, holding conditions, and sampling approaches, and work to develop standards. Equations (11)–(14) are an initial attempt to explore these influences (Fig. 3). They emphasize that equivalent changes in the number of infected animals, their shedding rates, the proportion of the environment sampled, or rates of degradation all have equivalent effects on the amount of analyte in a sample, and thus detection probability. It is worth noting, however, that researchers may have control over certain key variables, such as the duration and volume in which animals are held before collecting samples, allowing them to maximize sensitivity. For instance, if rates of eDNA shedding and degradation are fairly constant and fast relative to changes in the underlying infection intensity, one would expect eDNA concentrations in water to approach an equilibrium over time, at least as a first approximation (Eq. (13)). The rate at which it asymptotes depends on the rate of degradation, and so water quality, temperature, pH, etc. are likely to be important^78,79^. However, unless rates of degradation are exceedingly rapid, one would expect concentrations of eDNA to accumulate over several days and so investigators might increase sensitivity by holding animals longer prior to sampling.

## Discussion

While efficiency is not the only important criteria for a testing scheme (e.g., the expertise, specialized equipment, and costs involved must also be considered), routine pathogen surveillance and thus a “clean trade” becomes more feasible as the number of samples that must be analyzed are reduced. Pooling swabs and sampling eDNA are both, in principle, viable approaches to reducing sample sizes, although to much different extents and with different drawbacks in their use.

Pooling and batch-processing samples collected from individual animals (e.g., swabs) can reduce several fold the number of diagnostic tests (e.g., DNA extractions and PCR reactions) required to detect rare infections. While some *n* samples must still be collected, pooling them into groups of size *k* means that only *n*/*k* pools need be processed, which may substantially reduce the time and costs of surveillance. The critical question for pooling is the degree to which sensitivity, the probability of correctly identifying infections if present, declines as samples are pooled. Laurin *et al*.^74^ found that sensitivity generally declined with pooling in aquatic pathogens, in part because moderate concentrations of analyte (e.g., pathogen DNA) may be diluted below reliable detection limits in pools^75^, but there are few studies that properly evaluate this. However, given the quantitative nature of detection (Fig. 3) and the large amount of variation in infection intensities and analyte in a sample, it may not be possible to provide simple, general estimates of sensitivity when pooling^75^.

In the case of *Bsal*, Sabino-Pinto *et al*.^23^ found that diagnostic sensitivity of the typical qPCR assay^69^ was unaffected when DNA was extracted from pools of up to four skin swabs. While the authors specifically recommend against pooling in trade and quarantine settings given the risks associated with the failure to detect a single infected individual^23^, their results suggest pooling could reduce several-fold the number of DNA extractions and qPCR reactions required to achieve the EU’s requisite detection probability in a shipment (Figs. 1 and 2). Again, empirical tests in real-world settings, especially with low-level *Bsal* infections, are needed to evaluate the actual sensitivity when pooling samples. Hyatt *et al*.^80^, for instance, found that diagnostic sensitivity of their qPCR assay for *Bd* remained high in pools of five swabs, so long as “equivocal” results (one or two of three wells positive) were scored as positive—likely because of a dilution of target DNA—but even here, one low-level infection went undetected in a pool. Still, losses in sensitivity may be offset by increases in the fraction of a population or consignment that can be screened when samples are pooled, essentially increasing the chances that a rare infected individual is included in a sample. For instance, even if sensitivity is reduced from a probability of one with individual swabs to 0.75 with a pool of four swabs, many fewer assays need be run on the pools than the individual swabs to attain the same confidence of detecting an infection (Fig. 2). Equation (7) and the included R code can be used to evaluate this trade-off.

Environmental DNA offers a different trade-off. The samples collect genetic material shed into the water (or environment more broadly), so whatever material is shed, which may be minimal, is diluted in a larger volume and might be degraded. All of which means that fairly little target DNA may end up in any given eDNA sample. Thus, one would expect the diagnostic sensitivity of eDNA samples to be quite low relative to individual-based samples^26,73^ (Fig. 3). However, eDNA samples from the *entire* population, at least in the context of small captive populations or consignments of animals in water, perhaps with the aid of mechanical homogenization. This allows eDNA to circumvent the problem of including rare infected individuals in sample that is intrinsic to individual-level sampling (and pooling). As a result, even with low sensitivity, eDNA sampling can be much more efficient at detecting infections in large consignments than perfectly sensitive individual-based samples or pools of samples. Moreover, sample sizes required to detect a rare infection do not increase with population or consignment size (Fig. 2). Sampling eDNA thus offers the promise of dramatically reducing the amount of sampling required to ensure disease freedom in the trade of live aquatic and semi-aquatic animals.

The actual performance of eDNA-based detection likely depends on the nature of the consignment (e.g., volume, time animals have spent in water), collection and processing (e.g., volume filtered, presence and removal of PCR inhibitors), and biological realities (e.g., rates of shedding and degradation). It is important to evaluate these factors empirically so that the results can be properly interpreted. The simple model presented in Eqs. (12)–(14) provides a useful starting point, illustrating how eDNA concentrations, and thus per sample detection probabilities, vary with volume, time, and rates of shedding and degradation (Fig. 3). The proportion of holding water sampled and time animals spend in the water, shedding pathogen eDNA, are likely key parameters, but may vary substantially among taxa, life stages, and settings. A clever investigator, however, could use these factors to increase the sensitivity of eDNA-based samples, for instance by holding animals in small volumes of clean water for longer periods of time to ensure that target eDNA accumulates to readily detectable levels before collecting eDNA samples.

Perhaps the most fundamental question is whether pathogen eDNA is swamped by non target host or microbial DNA or PCR inhibition increases with population size. While not unique to eDNA—PCR inhibition is common with swab-based detection of chytrid fungi—the issue maybe be magnified for environmental samples. If swamping or inhibition are a problem then the number of eDNA samples required to attain a particular detection probability would need to increase with population size and Eq. (9) adjusted accordingly. I must stress that it is only with a clear understanding of how these manifold conditions influence detection probabilities^78^ can eDNA-based detection be used reliably in the variety of settings and types of consignments that comprise the live animal trade.

The goal of developing a “clean trade” may be the most realistic strategy for preventing the movement and introduction of pathogens such as *Bsal* into new areas and naive hosts. But it is a daunting task given the immense number of animals involved in international and regional trade. The formulae developed here place sample pooling and eDNA in closed populations on a firm theoretical foundation. They suggest that pooling individual-level samples and, especially, collecting eDNA can substantially reduce sample sizes required to ensure rare, but important infections are found. Moreover, sampling at key nodes in the distribution networks or crucial segments of the live animal trade (e.g., particular species, sources) can make surveillance even more tractable^10^. It is my hope that these approaches can help bring clean trade into reach for a greater number of taxa, places, and contexts.

## Equations

### Individual-level sampling

The probability of obtaining *x* infected individuals (*D*^+^) in a sample of size *n* taken without replacement from a population of size *N*, of which *d* are infected is described by a hypergeometric distribution^81^:

1 $$P({D}^{+}=x)=\frac{(\begin{array}{c}d\\ x\end{array})(\begin{array}{c}N-d\\ n-x\end{array})}{(\begin{array}{c}N\\ n\end{array})}.$$

However, diagnostic tests are rarely perfect. Infected individuals are correctly detected with probability *Se* (diagnostic sensitivity) and uninfected animals correctly test negative with probability *Sp* (diagnostic specificity); false negatives and false positives occur with probabilities 1 − *Se* and 1 − *Sp*, respectively. Cameron and Baldock^81^ extended the hypergeometric model to include imperfect tests, such that the probability of observing *x* positive tests (*T*^+^) is:

2 $$P({T}^{+}=x)=\mathop{\sum }\limits_{y=0}^{d}\,\left(\frac{(\begin{array}{c}d\\ y\end{array})(\begin{array}{c}N-d\\ n-y\end{array})}{(\begin{array}{c}N\\ n\end{array})},\times ,\mathop{\sum }\limits_{j=0}^{min(x,y)},\,,[(\begin{array}{c}y\\ j\end{array})S{e}^{j}{(1-Se)}^{y-j}\times (\begin{array}{c}n-y\\ x-j\end{array}){(1-Sp)}^{x-j}S{p}^{n-x-y+j}]\right).$$

Often our goal is to determine the probability of at least one positive sample, given particular parameter values. Consider a consignment of *N* = 100 salamanders, some 3% (*d* = 3) of which are infected, screened with individual swabs with a diagnostic sensitivity of *Se* = 0.8, matching the assumption in the EU regulations^70^, and perfect specificity (*Sp* = 1). We see that the probability of detecting at least one of these infections is, using the equivalence *P*(*T*^+^ ≥ *x*) = 1 − *P*(*T*^+^ = 0):

3 $$P({T}^{+}\ge 1)=1-\mathop{\sum }\limits_{y=0}^{3}\,\left(\frac{(\begin{array}{c}3\\ y\end{array})(\begin{array}{c}100-3\\ n-y\end{array})}{(\begin{array}{c}100\\ n\end{array})}\times \mathop{\sum }\limits_{j=0}^{{\rm{\min }}(x,y)}[(\begin{array}{c}y\\ j\end{array}){0.8}^{j}{(1-0.8)}^{y-j}]\right).$$

Note that the part involving specificity simplifies to 1 when *Se* = 1. Because *x* = 0, *j* is always zero and so this simplifies to:

4 $$P({T}^{+}\ge 1)=1-\mathop{\sum }\limits_{y=0}^{3}\,\left(\frac{(\begin{array}{c}3\\ y\end{array})(\begin{array}{c}97\\ n-y\end{array})}{(\begin{array}{c}100\\ n\end{array})},\times ,{(1-0.8)}^{y}\right)$$

5 $$\,=1-\left[\frac{(\begin{array}{c}3\\ 0\end{array})(\begin{array}{c}97\\ n-0\end{array})}{(\begin{array}{c}100\\ n\end{array})},\times ,{0.2}^{0},+,\frac{(\begin{array}{c}3\\ 1\end{array})(\begin{array}{c}97\\ n-1\end{array})}{(\begin{array}{c}100\\ n\end{array})},\times ,{0.2}^{1},+,\frac{(\begin{array}{c}3\\ 2\end{array})(\begin{array}{c}97\\ n-2\end{array})}{(\begin{array}{c}100\\ n\end{array})},\times ,{0.2}^{2},+,\frac{(\begin{array}{c}3\\ 3\end{array})(\begin{array}{c}97\\ n-3\end{array})}{(\begin{array}{c}100\\ n\end{array})},\times ,{0.2}^{3}\right].$$

One can then try values of *n* such that *P*(*T*^+^ ≥ 1) ≥ 0.95 or any other threshold surveillance sensitivity. In this scenario the threshold is met when *n* ≥ 79. While this calculation can be done by hand, it becomes quite tedious. Using the R code found below the same calculation can be achieved with pooledHyp_Atleast1(d=3, N=100, m=79, k=1, Se=0.8, Sp=1). Note that that this function is written for pooled samples (see next subsection), but we can use it for individual samples with *m* = *n* “pools” (=samples) of size *k* = 1.

### Pooling individual-level samples

When the *n* samples are divided into *m* groups of size *k* = *n*/*m*, which are then tested as pools, the probability that *x* of the *m* pools contain an infected individual in them (*Pool*^+^) is described by the “pooled hypergeometric” of Theobald and Davies^24^:

6 $$P(Poo{l}^{+}=x)=(\begin{array}{c}m\\ x\end{array})\mathop{\sum }\limits_{i=0}^{x}\,{(-1)}^{i}(\begin{array}{c}x\\ i\end{array})\frac{(\begin{array}{c}N-d\\ (m-x+i)k\end{array})}{(\begin{array}{c}N\\ (m-x+i)k\end{array})},$$

for max[0, *m* − (*N* − *d*)/*k*] ≤ *x* ≤ min(*m*, *d*).

This formula can be extended to include false negatives and false positives in a manner analogous to Cameron and Baldock^81^, where the probability of observing *x* positive tests (${T}_{pool}^{+}$) for the *m* pools is:

7 $$\begin{array}{l}P({T}_{pool}^{+}=x)\\ \qquad=\mathop{\sum }\limits_{y=0}^{\min(m,d)}\,\left(\left(\begin{array}{c}m\\ y\end{array}\right)\mathop{\sum }\limits_{i=0}^{y}\,{(-1)}^{i}\left(\begin{array}{c}y\\ i\end{array}\right)\frac{\left(\begin{array}{c}N-d\\ (m-y+i)k\end{array}\right)}{\left(\begin{array}{c}N\\ (m-y+i)k\end{array}\right)}\,\right.\\ \qquad\left. \times \mathop{\sum }\limits_{j=0}^{\min(x,y)}\,\left[\left(\begin{array}{c}y\\ j\end{array}\right)S{e}^{j}{(1-Se)}^{y-j}\left(\begin{array}{c}m-y\\ x-j\end{array}\right){(1-Sp)}^{x-j}S{p}^{m-x-y+j}\right]\right)\end{array}$$

It is important to note that this formulation assumes diagnostic sensitivity and specificity are not influenced by the composition of a pool. A pool is equally likely to test positive if one or all of the individuals within it are positive, or if the pool is comprised of few or many samples. This assumption may be questionable for large pools, but may be reasonable with small *k*.

Returning to the example of a consignment of *N* = 100 with *Se* = 0.8 and *Sp* = 1, but now pooling swabs into groups of size *k* = 4 and assuming that the *m* pools is greater than the *d* = 3 infections, we can find the probability of at least one pool testing positive as:

8 $$P({T}_{pool}^{+}\ge x)=1-P({T}^{+}=0)=1-\mathop{\sum }\limits_{y=0}^{3}\,\left((\begin{array}{c}m\\ y\end{array}),\mathop{\sum }\limits_{i=0}^{y},\,,{(-1)}^{i},(\begin{array}{c}y\\ i\end{array}),\frac{(\begin{array}{c}97\\ (m-y+i)4\end{array})}{(\begin{array}{c}100\\ (m-y+i)4\end{array})},\times ,{(1-0.8)}^{y}\right),$$

but with the double summation this is rather unwieldy to solve by hand. Rather one can use the code, pooledHyp_Atleast1(d=3, N=100, m=20, k=4, Se=0.8, Sp=1), to find that *P*(*T*^+^ ≥ 1) ≥ 0.95 when *m* ≥ 20.

### Environmental DNA

Let us assume that target eDNA is well mixed in the water. We can then use a binomial to describe the distribution of positive eDNA tests (${T}_{eDNA}^{+}$) because taking one sample does not affect the probability of detection in subsequent samples. Retaining the definition of sensitivity as the probability of detecting one infection if present (i.e., $Se=P({T}_{eDNA}^{+}|d=1)$), we obtain the following expression for the probably of observing *x* positive samples:

9 $$P({T}_{eDNA}^{+}=x)=(\begin{array}{c}n\\ x\end{array}){[1-{(1-Se)}^{d}+{(1-Se)}^{d}(1-Sp)]}^{x}{[{(1-Se)}^{d}Sp]}^{n-x}.$$

Note that because each eDNA sample collects material from all *d* infected individuals in the population, success is defined in the binomial as the probability of a positive eDNA sample [1 − (1 − *Se*)^*d*^], ignoring false positives, rather than the probability a sample includes an infected individual, as is the case when using the binomial to describe detection probabilities with individual samples taken with replacement. Multiple infected individuals in a population simply increase the amount of target eDNA to be detected and thus the effective sensitivity of the eDNA test. Both sensitivity and specificity are assumed to be constant and independent of the *N* − *d* uninfected animals in the population.

Finding the number of replicate eDNA samples to ensure $P({T}_{eDNA}^{+}\ge 1)\ge 0.95$ is rather simpler in this model. If we assume there are *d* = 3 infected individuals in a consignment (the size of which does not enter the equation, and a very low sensitivity, *Se* = 0.05, but perfect specificity, *Se* = 1, we can the probability that at least one sample tests positive:

10 $$P({T}_{eDNA}^{+}\ge 1)=1-P({T}_{eDNA}^{+}=0)=1-{[{(1-0.05)}^{3}]}^{n}.$$

Notice that both the binomial terms and the first term in brackets simplify to one since *x* = 0; in other words, we only need to subtract the probability that *all n* samples test negative from one. $P({T}_{eDNA}^{+}\ge 1)\ge 0.95$ when *n* ≥ 20. While this is simple to calculate by hand, the code, eDNA_Atleast1(d=3, m=20, Se=0.05, Sp=1) produces the same result.

### The quantitative nature of detection

Any test that reacts directly with a pathogen (e.g., pathogen DNA or antigens) or host responses (e.g., antibodies) is necessarily dose-dependent. Focusing on DNA-dependent detection methods (e.g., PCR), let us assume that every copy, *C*, of the target DNA sequence in a sample has a small, fixed probability of causing a positive result in a reaction, *ϕ*. The probability of a positive test result can then be described by a “single-hit” model^82,83^:

11 $$P({T}^{+})=1-{e}^{-\phi C},$$

which results in a typical dose-response relationship (Fig. 3). (A logistic relationship between *P*(*T*^+^) and log(*C*), which is commonly used in studies of analytic sensitivity, yields similar results, but requires an extra parameter.) If the *C* copies come from a single infected individual, then this function describes diagnostic sensitivity. The magnitude of *C* in a sample can therefore have a strong influence on sensitivity. As noted in the main text, one would expect greater variation in *C* with eDNA sample than with individual-level samples because eDNA is not collected directly from an animal, but from the environment, which can also play a role (although external swabs are also influenced by the environment). A simple model is useful in clarifying the factors that likely determine the number of target copies in an eDNA sample.

In this model, the number of copies of target eDNA in a sample increase as the *d* infected individuals shed into the water at rate *ψ*, some portion, *α*, of which ends up in each eDNA sample, and decrease as eDNA degrades at rate *δ*:

12 $$\frac{dC}{dt}=\psi d\alpha -\delta C.$$

The solution is:

13 $$C(t)={C}_{0}{e}^{-\delta t}+\frac{\psi d\alpha }{\delta }(1-{e}^{-\delta t}),$$

where *C*_0_ is the initial concentration in the water (the first term on the right-hand side is zero when *C*_0_ = 0). The solution asymptotes to C^*^ = *ψdα*/*δ* over time at a rate dependent upon *δ*. Substituting the equilibrium solution into the single-hit model (Eq. (11)), one can see that detection probability depends on these rates in a straightforward way:

14 $$P({T}_{eDNA}^{+})=1-{e}^{-\phi {C}^{\ast }}=1-{e}^{-\phi \frac{\psi d\alpha }{\delta }}.$$

While this model has several terms, they have clear biological meaning and could be estimated from experimental data^84–86^. Moreover, they enter the model as a product, with equivalent effects, at least at equilibrium. That is, doubling the volume of water collected in an eDNA sample is the same as doubling the number of infected individuals or halving the decay rate. This model also provides a way to link the many factors that can affect eDNA-based detection via their influence on target copy number, *C* (e.g., degradation, *δ*, is likely temperature-dependent^86^), or PCR efficiency (e.g., *ϕ* might be a function of pH and environmental inhibitors^29^).

### R code for sample size calculations

The pooled hypergeometric distribution of Theobald and Davies^24^ can be implemented in R using the “big integer” versions of multiplication and the binomial expansion found in the gmp package^87^ for greater precision:

pooledHyper <− function(x, d, N, m, k){

i = 0:x

as.numeric(

gmp::mul.bigq(gmp::chooseZ(m, x),

sum((−1)^i * gmp::chooseZ(x,i) * gmp::chooseZ(N-d, (m-x+i)*k)/

gmp::chooseZ(N, (m-x+i)*k)

)

)

)

}

This is then extended to include sensitivity and specificity, as in Eq. (7):

px_pooledHyper<− function(x, d, N, m, k, Se, Sp,…) {

# check inputs

if(x>m) stop(“x is greater than m (number of pools)”)

if(m*k>N) stop(“individuals sampled (m*k) is greater than population size (N)”)

stopifnot(Se<=1, Se>=0, Sp<=1, Sp>=0, length(x)==1)

innerSums <− 0

for(y in 0:m){

j<− 0:min(y,x)

innerSums[y+1] <- pooledHyper(x=y, d=d, N=N, m=m, k=k) *

sum(dbinom(j, size=y, prob=Se) * dbinom(x-j, size=m-y, prob=(1-Sp)))

}

sum(innerSums) # return sum of inner summations

}

Note that when k=1 the “pools” are individual samples, as in Eq. (6). A wrapper function can then be used to find the probability of at least one sample or pool of samples testing positive:

pooledHyp_Atleast1<− function(d, N, m, k, Se, Sp,…){

1-px_pooledHyper(x=0, d=d, N=N, m=m, k=k, Se=Se, Sp=Sp)

}

Equation (9) for eDNA can be implemented in R as:

px_eDNA <− function(x, d, n, Se, Sp){

# check inputs

stopifnot(Se<=1, Se>=0, Sp<=1, Sp>=0)

choose(n,x) * (1 - (1-Se)^d+((1-Se)^d)*(1-Sp))^x * (((1-Se)^d)*Sp)^(n-x)

}

A wrapper function is then used to find the probability of at least one eDNA samples testing positive:

eDNA_Atleast1<− function(d, m, Se, Sp=1,…){

1-px_eDNA(x=0, d=d, n=m, Se=Se, Sp=Sp)

}

## Data Availability

No datasets were generated or analysed during the current study.
